# Diagnostic delay in extrapulmonary tuberculosis and impact on patient morbidity: A study from Zanzibar

**DOI:** 10.1371/journal.pone.0203593

**Published:** 2018-09-06

**Authors:** Melissa Davidsen Jørstad, Jörg Aẞmus, Msafiri Marijani, Lisbet Sviland, Tehmina Mustafa

**Affiliations:** 1 Department of Thoracic Medicine, Haukeland University Hospital, Bergen, Norway; 2 Centre for International Health, Department of Global Public Health and Primary Care, University of Bergen, Bergen, Norway; 3 Centre for Clinical Research, Haukeland University Hospital, Bergen, Norway; 4 Department of Diagnostic Services, Mnazi Mmoja Hospital, Zanzibar, The United Republic of Tanzania; 5 Department of Clinical Medicine, Faculty of Medicine, University of Bergen, Bergen, Norway; 6 Department of Pathology, Haukeland University Hospital, Bergen, Norway; Central University of Tamil Nadu, INDIA

## Abstract

**Background:**

Early and proper treatment of tuberculosis could have an important impact on the morbidity, mortality and the economic situation of patients. There is insufficient knowledge on the extent of diagnostic delay and the associated factors in extrapulmonary tuberculosis (EPTB). The aims of this study were to assess the health care seeking behaviour, EPTB knowledge and diagnostic delay in presumptive EPTB patients at the main referral hospital in Zanzibar, factors associated with longer delay, and the impact of untreated EPTB on self-rated health.

**Materials and methods:**

Prospective data collection using a semi-structured questionnaire in patients presenting with symptoms suggestive of EPTB. The time between the onset of symptoms and first visit to a health care provider (patient delay), and then to the initiation of treatment (health system delay) and total delay were analysed according to sociodemographic and clinical factors and health care seeking trajectories. The EQ-5D-3L was used among the adult EPTB patients to assess the impact of treatment on self-rated health.

**Results:**

Of the 132 patients with median age of 27 years (interquartile range 8–41), 69 were categorized as TB cases and 63 as non-TB cases. The median patient, health system and total delays were 14, 34 and 62 days respectively, among the EPTB patients. A longer health system delay with repeated visits to the same health care level was reported. Significantly better self-rated health status was described after treatment. The knowledge regarding extrapulmonary disease was low.

**Conclusion:**

Many EPTB patients, presenting to the main referral hospital in Zanzibar, experience a long delay in the initiation of treatment, specially patients with TB lymphadenitis. The health system delay is the major contributor to the total delay. The improvement of self-rated health after treatment implies that timely treatment has the potential to reduce morbidity and the economic loss for the patient.

## Introduction

Tuberculosis (TB) continues to be a major global public health problem. In 2015, the World Health Organization (WHO) estimated that there were 10.4 million incident cases of TB worldwide [1]. Of the notified new cases, extrapulmonary TB (EPTB) accounted for 15% of the cases [1]. The proportion of EPTB is higher in females [2–6], people with African or Asian origin [2–4], TB and human immunodeficiency virus (HIV) co-infected patients [2, 3, 7] and at younger ages [2–6].

Timely detection and proper treatment of TB are two of the key elements of an effective TB control programme [8]. In the context of the WHO`s End TB strategy, which calls for early diagnosis and treatment of all TB cases [9], the detection, treatment and follow-up of EPTB cases should also be given priority. Diagnosing EPTB is challenging as it frequently has non-specific clinical presentation and may simulate other conditions, which may contribute towards delay in diagnosis. In addition, lack of rapid, simple and accurate diagnostic tools for diagnosing EPTB may prolong diagnostic delay [10]. Since EPTB is rarely infectious the aspect of transmission is not as important as in pulmonary TB (PTB) patients, but delay in diagnosis and treatment could lead to increased disease severity, more complications and economic costs for the patient and the families affected.

Most studies on the diagnostic delay in TB have focused on PTB and adults [11–13]. Fewer studies have included children and EPTB patients, and these studies have reported longer delays among EPTB patients as compared to PTB [14–17]. However, there is insufficient knowledge on the extent of the various delays and the associated factors among EPTB patients. It is important to identify factors contributing to the distinct types of delays in EPTB, which could provide information for evidence-based intervention to improve case-finding and prompt diagnosis and treatment.

The United Republic of Tanzania is among the 30 high TB burden countries in the world [1]. Zanzibar, a semi-autonomous part of the United Republic of Tanzania, comprising the two main islands Unguja and Pemba and some smaller islands, has 1.3 million inhabitants [18]. According to a national TB prevalence survey in 2012, the estimated prevalence of bacteriologically confirmed PTB in Zanzibar was 124/100 000 in the adult population [19]. Among the 814 notified incident TB cases in Zanzibar in 2015, 195 (24%) were EPTB cases [20]. Zanzibar has 100% directly observed treatment coverage, however, the notified TB cases are still below the estimated numbers of TB cases.

The aims of this study were to assess the health care seeking behavior, EPTB knowledge and diagnostic delay in presumptive EPTB patients presenting at the Mnazi Mmoja Hospital (MMH), Zanzibar, the factors associated with longer delay and the impact of untreated EPTB on self-rated health status of patients. The presumptive EPTB cases were categorized into TB and non-TB cases based on a composite reference standard allowing us to compare the TB and non-TB cases with similar clinical presentation.

## Materials and methods

### Study setting

The study was conducted at MMH, which is the main referral hospital in Zanzibar. The public health care system in Zanzibar is divided into three levels [21]. The primary level consists of primary health care units (PHCU), PHCU+, which are supposed to provide additional services of delivery, dental, dispensing and laboratory, and primary health care centres/cottage hospitals (PHCC) which serve as referral level for PHCU and PHCU+. PHCCs has both in- and outpatient services, average capacity of 30 beds, and provides additional services such as diagnostic imaging (ultrasound and x-ray). The secondary and tertiary level consist of district hospitals, special and referral hospitals. In 2013, there were 100 PHCUs, 34 PHCU+, 4 PHCCs, 3 district hospitals, 2 special hospitals and 1 main referral hospital (MMH) in Zanzibar [21]. MMH, situated in the capital at Unguja Island, provides primary and secondary health care for some districts, in addition to tertiary health care. There is a good distribution and access to primary health care services, with 95% of the population living within 5 km to the nearest public health facility [21]. However, there is a limited capacity to perform invasive procedures for diagnostic purposes at the primary level of health care. MMH is the only public hospital with the capacity to perform the diagnostic cytological/histological evaluation of fine-needle aspirates and biopsies. In 2013/14, there were 4 private hospitals, all situated at Unguja Island, 71 private clinics/dispensaries, 25 pharmacies and 335 over-the-counter drug shops in Zanzibar [22]. The private health facilities are predominantly located in the major towns. The TB preventive, diagnostic and treatment services are integrated into the public health service at all levels. Anti-TB treatment (ATT) is free of charge and all public health facilities and some private facilities are providing TB treatment services. TB/HIV collaborative activities are well-organized, and all TB patients are offered HIV testing and counselling.

### Study design and population

The study participants were prospectively enrolled at MMH, from 1^st^ of August 2014 until 31^st^ of August 2015, from outpatient departments and the hospital wards. The study population was patients of all ages presenting with symptoms suggestive of EPTB. The current study was part of a larger study evaluating the implementation of a new diagnostic test for the diagnosis of EPTB at MMH [23], and eligible patients were included if a representative biological specimen was sampled for laboratory investigations from the presumptive site of EPTB infection. Those who did not give an informed written consent or had received ATT during the previous year were excluded. All study participants were interviewed at the time of inclusion using a pretested semi-structured questionnaire and closely followed until a diagnosis was concluded by the local clinicians. Additional visits to health care providers after inclusion in the study were documented. Patients starting ATT were further assessed at 2–3 months and at end of ATT to evaluate response to treatment, and patients not starting ATT were followed until recovery or until a diagnosis was established. At the end of the study, patients were categorized as TB cases or non-TB cases using a composite reference standard, including clinical signs and symptoms, radiological findings, results from various laboratory investigations, response to specific non-tuberculous therapy and response to ATT [23].

### Data collection and outcomes

The main outcomes were patient, health system and total delays in days. Additionally, we assessed general TB knowledge and EPTB knowledge among all the study participants and self-rated health status in adult EPTB patients before ATT and after the completion of ATT.

The following definitions were used. The onset of symptoms referred to the time at which the first symptom appeared, either localized symptoms or constitutional symptoms which led to the care-seeking. The patient delay was defined as the time interval between the onset of symptoms and the patients`first visit to a health care provider because of those symptoms. The health system delay was defined as the time interval between the patients`first visit to a health care provider and the initiation of ATT. The total delay was defined as the sum of patient delay and health system delay. Health care providers were defined as modern health facilities such as private clinics/dispensaries, PHCU, PHCU+, PHCC or hospitals owned by the government or the private sectors. Registered pharmacies were also included, whereas, non-formal health providers, such as traditional healers and over-the-counter drug stores were not included in this definition.

A semi-structured questionnaire was developed in English and translated to the local language, Swahili. Further, the Swahili version was translated back to English by a person who had not seen the original version. The two English versions were compared to evaluate the validity of the questions. To adjust the language to the local setting, two local bilingual individuals assessed the questions prior to testing the questionnaire among inpatients (*n* = 3) at MMH to clarify confusing questions and estimating the required time for filling in the questionnaire. Two local medical officers, who underwent training, conducted all the interviews. Included in the questionnaire were questions related to sociodemographic characteristics, symptoms and their duration, estimation of the time interval from the onset of symptoms until the first contact with a health care provider, number of different health care providers contacted due to the current illness and other questions regarding medical history, health care seeking behavior and TB knowledge [23]. The date of starting ATT and medical history were counterchecked from patient medical records and TB treatment cards.

The study participants`TB knowledge was assessed by questions related to symptoms, site of infection, transmission and treatment of TB (S1 Table). A scoring system was designed based on these questions and the median score among all the respondents was used as a cut-off point to dichotomize the responses into two categories, lesser (below the median) and higher (equal or above the median) TB knowledge. The TB knowledge questions were answered by the parent/guardian for study participants < 15 years of age.

In addition to the questionnaire, the adult patients starting ATT answered the EQ-5D 3 level version (EQ-5D-3L) [24], before and after ATT, to provide a simple descriptive health profile on self-rated health. The EQ-5D-3L comprises the EQ-5D descriptive system and the EQ visual analogue scale. The EQ-5D descriptive system consists of 5 dimensions (mobility, self-care, usual activities, pain/discomfort and anxiety/depression) with 3 levels (level 1, no problems; level 2, some problems; level 3, extreme problems). The EQ VAS comprises a vertical, visual analogue scale, with endpoints labelled “Best imaginable health state”, marked 100, and “Worst imaginable health state”, marked 0, and records the respondent`s self-rated health at the time of completion. The EQ-5D-3L was provided in the local language (EuroQoL Group 1990).

The exposure variables included age, sex, educational level, housing, HIV status, site of TB disease, self-treatment before care-seeking, distance to the nearest health care provider, initial health care provider visited after the onset of symptoms, antibiotics given at first visit, number of visits to health care providers, and TB knowledge.

### Statistical analysis

Descriptive statistics were presented, and group differences were compared using the Chi-square test for proportions. Differences in the delay variables were assessed by Mann-Whitney or Kruskal-Wallis tests for differences between the groups as well as Wilcoxon signed rank test for repeated measures. The general significance level was set to 0.05. Due to the larger number of tests we had to consider multiple testing effects. We used the Bonferroni adjustment, leading to the marginal levels of 0.0083 (6 tests) for sociodemographic and clinical characteristics, 0.01 (5 tests) for first health care provider visited, 0.0042 (12 tests) for the delay, and 0.0125 (4 tests) for TB knowledge score. Data analysis was performed using SPSS 24 (Armonk, NY) and graphics was created using Matlab 9.0 (Natick, MA).

### Ethical considerations

Ethical clearance was obtained from the Regional Committee for Medical and Health Research Ethics, Western-Norway (REK Vest) and the Zanzibar Medical Research and Ethics Committee (ZAMREC). All study participants signed an informed written consent. In patients < 18 years, the written consent was signed by the parent/guardian, in addition, patients ≥ 7 years had to sign the consent form.

## Results

### Sociodemographic and clinical characteristics

Of 146 eligible patients presenting with presumptive EPTB, 132 patients were enrolled in the study and 14 excluded (5 refused to participate, 3 on treatment within the last year, 6 with no representative sample for laboratory investigation). Sixty-nine participants were categorized as TB cases, and 63 as non-TB cases. The sociodemographic and clinical characteristics of the included study participants are described in Table 1. The TB and non-TB patients did not differ significantly with regard to sex, residence, educational level, HIV status or presumptive site of infection. The median age was 27 years (interquartile range (IQR), 8–41 years). Most of the non-TB patients were either children or ≥ 45 years, whereas the majority of the TB patients were between 15–44 years. HIV status was known in 99 (75%) patients, and 22 (22%) of these were HIV positive.

**Table 1:** Sociodemographic and clinical characteristics of the study participants, n (%).

Characteristics		TB patients	non-TB patients	*P* value
		*n* = 69	*n* = 63	
Sex				.811
	Male	38 (55%)	36 (57%)	
	Female	31 (45%)	27 (43%)	
Age groups, years				.010
	0–14	18 (26%)	25 (40%)	
	15–44	40 (58%)	20 (32%)	
	≥45	11 (16%)	18 (29%)	
Residential status				.871
	Urban	48 (70%)	43 (68%)	
	Rural	21 (30%)	20 (32%)	
Highest educational level^a^				.592
	≤ primary school	26 (51%)	21 (57%)	
	> primary school	25 (49%)	16 (43%)	
Main household income^b^				
	Agriculture/fishing	19 (33%)		
	Governmental/private sector	15 (26%)		
	Self-employed	24 (41%)		
Housing^c^				
	Own house	37 (59%)		
	Renting/living with relatives	26 (41%)		
Number of people in household^d^				
	1–5	24 (38%)		
	6–10	25 (40%)		
	≥11	14 (22%)		
HIV status				.396^e^
	Negative	48 (75%)	29 (83%)	
	Positive	16 (25%)	6 (17%)	
	Unknown	5 (-)	28 (-)	
Presumptive site of infection^f^				.263
	Lymphadenitis	36 (52%)	33 (52%)	
	Pleuritis	20 (29%)	12 (19%)	
	Other sitesf	13 (18%)	18 (29%)	

**Abbreviations:** TB, tuberculosis; HIV, human immunodeficiency virus.

^a^ Only adult patients. Patients with missing values excluded (n = 1).

^b, c, d^ Patients with missing values excluded (n = ^b^; 11, ^c, d^; 6). Not recorded for non-TB patients.

^e^ Only comparing patients with known HIV serostatus.

^f^ TB patients; meningitis (n = 4), spondylitis (n = 1), pericarditis (n = 1), peritonitis (n = 7). Non-TB patients; meningitis (n = 6), osteomyelitis (n = 1), mastitis (n = 1), peritonitis (n = 10).

### Health care seeking behaviour and care seeking pathways

**Fig 1:**
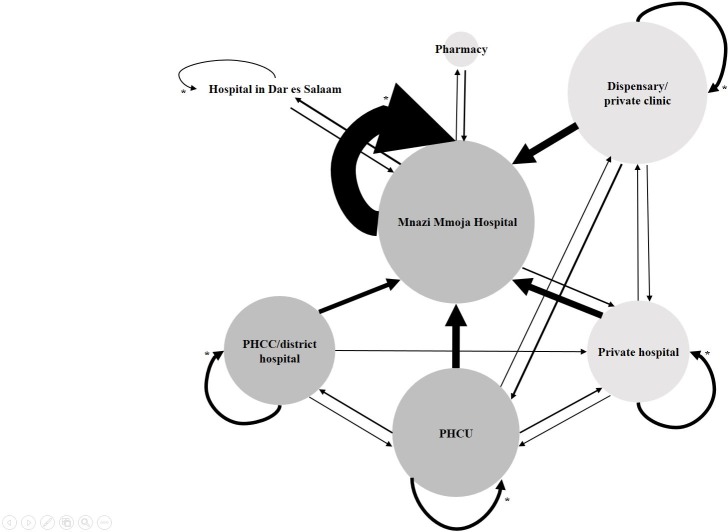
Health care seeking pathways in TB patients presenting at Mnazi Mmoja Hospital, Zanzibar. The size of the arrows shows the number of patients going in the direction of the arrow in the health care system. The size of the circles correlates to the number of patients visiting each respective site as the first place of contact (Mnazi Mmoja hospital, n = 21; private clinic/dispensary, n = 16; primary health care unit, n = 14; primary health care centre/district hospital, n = 9; private hospital, n = 8; pharmacy, n = 1). Colour—Dark grey, public health sector; light grey, private health sector. *Repeated visits at the same health care level, either same place or different health care provider at the same level. Abbreviations: PHCC, primary health care centre; PHCU, primary health care unit.

Table 2 shows the distribution of health care seeking trajectories among the TB and non-TB patients. The majority of patients consulted public health care providers as their first contact with the health care system after the onset of symptoms. Patients who first visited a public health care provider were similar to those who initially went to a private health care provider with regard to sex, age groups, residence, HIV status and TB category (TB or non-TB cases) (all *P* > .1). Further, no differences were found regarding the same variables when comparing those who contacted MMH first with patients who initially visited any health care provider other than MMH. Overall, 39 patients (30%) sought care at MMH as their first contact, while 32 (24%) at PHCUs, 22 (17%) at private clinics/dispensaries, 18 (14%) at PHCC/district hospitals, 18 (14%) at private hospitals and 2 (2%) at pharmacies. The practice of self-medication before contacting a health care provider was common and reported by 46% of the patients. When excluding patients only consulting MMH, 45/99 (45%) patients, reported repeated visits at the same level in the health care system before referral to MMH. Repeated visits to MMH before the commence of ATT were reported by 56/69 (81%) of the TB cases. Nearly half of the TB patients had > 3 visits to a health care provider, and 26% had visited ≥ 3 different health care providers, either at the same level or different level of health care, before the initiation of ATT. The most common care-seeking pathways among TB patients were; 18 (26%) patients had only visited MMH, 14 (20%) private clinic/dispensary and MMH, 9 (13%) private hospital and MMH, 9 (13%) PHCU and MMH, and 7 (10%) patients had been in contact with PHCC/district hospital and MMH. The patients were also asked to report if they had visited a traditional healer during the care-seeking interval. Only 7/69 (10%) of the TB patients reported visiting a traditional healer, 6 of these patients were females and all were ≥ 15 years. An illustration of health care seeking pathways among the TB cases is presented in Fig 1.

**Table 2:** Distribution of health care seeking trajectories and TB knowledge, n (%).

Variable		TB patients	non-TB patients	*P* value
		*n* = 69	*n* = 63	
Self-treatment before care seeking^a^				.959
	Yes	31 (46%)	29 (46%)	
	No	37 (54%)	34 (54%)	
Distance to nearest HCP (time)^b^				
	< 30 min	15 (23%)		
	30–60 min	32 (49%)		
	> 60 min	18 (28%)		
First HCP visited^c^				.281
	Public	44 (64%)	45 (73%)	
	Private	25 (36%)	17 (27%)	
First HCP visited^c^				.861
	MMH	21 (30%)	18 (29%)	
	HCP other than MMH	48 (70%)	44 (71%)	
Antibiotics given at first visit^d^				.869
	Yes	38 (58%)	36 (59%)	
	No	28 (42%)	25 (41%)	
Given a diagnosis prior to interview^e^				.672
	No	36 (59%)	38 (66%)	
	Yes, other diagnosis than tuberculosis	21 (34%)	18 (31%)	
	Yes. Tuberculosis	4 (7%)	2 (3%)	
Number of different HCP^f^				
	1	18 (26%)	14 (23%)	
	2	33 (48%)	28 (45%)	
	3	11 (16%)	13 (21%)	
	≥4	7 (10%)	7 (11%)	
Number of visits to HCP^g^				
	1	2 (3%)	5 (9%)	
	2	10 (14%)	14 (25%)	
	3	24 (35%)	19 (33%)	
	4	7 (10%)	8 (14%)	
	≥5	26 (38%)	11 (19%)	
TB knowledge^h^				.607
	Good (≥median)	38 (56%)	38 (60%)	
	Poor (<median)	30 (44%)	25 (40%)	
EPTB knowledge^h^				
	Yes	1 (1%)	0 (-)	
	No	67 (99%)	63 (100%)	

**Abbreviations:** TB, tuberculosis; HCP, health care provider; MMH, Mnazi Mmoja Hospital; EPTB, extrapulmonary tuberculosis.

^a^ Patients with missing values excluded (n = 1)

^b^ Patients with missing values excluded (n = 4). Not recorded for non-TB patients.

^c, d, e^ Patients with missing values excluded (n = ^c^; 1, ^d^; 5, ^e^; 13).

^f, g^ Patients with missing values excluded (n = ^f^;1, ^g^; 6). No comparison between TB patients and non-TB patient because the number of various health carer providers visited and the number of visits for TB patients were recorded until starting antituberculosis treatment, but for non-TB patients only to the day of the interview.

^h^ Patients with missing values excluded (n = 1).

### Diagnostic delay and associated factors

The patient, health system and total delays observed in TB patients are described according to different variables in Table 3. The median patient delay was 14 days (TB cases IQR, 5–28 days; non-TB cases IQR, 6–28 days) with no significant difference between TB and non-TB cases (*P* = .794). Patients with TB lymphadenitis reported a significantly longer patient delay compared to patients with other sites of TB disease. Interestingly, the median patient delay for those who first consulted MMH was 28 days compared to 11 days for those who initially visited other health facilities, but the difference was not statistically significant.

**Table 3:** Patient delay, health system delay and total delay among TB patients by sociodemographic and clinical variables and health care seeking trajectories^a^.

Variable		Patient delay (days)			Health system delay (days)			Total delay (days)		
		*n*	median (IQR)	*P* value	*n*	median (IQR)	*P* value	*n*	median (IQR)	*P* value
Sex				.400			.235			.777
	Male	38	13 (5–28)		36	36 (20–106)		36	58 (29–151)	
	Female	30	18 (6–47)		29	28 (14–67)		29	69 (31–118)	
Age groups, years				.674			.585			.137
	0–14	18	10 (5–28)		17	28 (22–65)		17	36 (28–113)	
	15–44	40	18 (4–42)		39	41 (16–115)		39	72 (34–163)	
	≥45	10	11 (7–26)		9	23 (16–61)		9	34 (26–94)	
Educational level^b^				.831			.032*			.297
	≤primary school	25	17 (4–79)		23	23 (11–51)		23	61 (31–108)	
	>primary school	25	16 (6–28)		25	48 (22–118)		25	91 (33–171)	
Housing				.253			.318			.031*
	Own house	36	13 (4–21)		35	28 (21–51)		35	41 (28–96)	
	Renting/living with relatives	26	21 (5–47)		26	59 (16–118)		26	98 (54–167)	
HIV status				.734^c^			.055^c^			.727^c^
	Negative	48	15 (5–36)		46	42 (21–110)		46	64 (30–155)	
	Positive	15	17 (5–41)		14	21 (10–67)		14	67 (31–121)	
	Unknown	5	8 (5–16)		5	28 (21–43)		5	36 (31–53)	
Site of TB infection				< .001**			.091			< .001**
	Lymphadenitis	35	28 (14–76)		34	55 (22–118)		34	99 (62–189)	
	Pleuritis	20	6 (4–11)		19	28 (16–48)		19	34 (20–65)	
	Other sites	13	6 (3–17)		12	22 (16–42)		12	30 (26–60)	
Self-treatment before care seeking				.870			.962			.903
	Yes	30	18 (4–42)		28	39 (17–80)		28	61 (30–141)	
	No	37	11 (6–28)		36	27 (20–79)		36	68 (30–127)	
First HCP visited				.025*			.001**			.445
	MMH	21	28 (8–111)		19	21 (7–34)		19	91 (31–124)	
	Other than MMH	47	11 (4–21)		46	46 (23–111)		46	61 (28–133)	
Antibiotics given at first visits							.141			
	Yes		-		35	41 (21–118)			-	
	No		-		27	24 (17–60)			-	
Distance to nearest HCP (time)				.553			.500			.993
	< 30 min	15	7 (6–38)		15	28 (16–86)		15	72 (21–179)	
	30–60 min	32	14 (4–28)		31	41 (23–86)		31	61 (30–129)	
	< 60 min	17	17 (6–67)		17	23 (14–56)		17	61 (30–124)	
Number of visits to HCP							< .001**			
	≤3		-		34	21 (12–29)			-	
	>3		-		31	69 (41–138)			-	
TB knowledge				.930			.280			.531
	Good (≥median)	37	14 (5–28)		35	28 (16–70)		35	62 (29–112)	
	Poor (<median)	30	14 (5–47)		29	41 (21–113)		29	65 (31–184)	

**Abbreviations:** TB, tuberculosis; IQR, interquartile range; HIV, human immunodeficiency virus, HCP, health care provider; MMH, Mnazi Mmoja Hospital

^a^ Patients with missing values excluded

^b^ Only adult patients included.

^c^ Only comparing patients with known HIV serostatus

* *P* value < 0.05

** Statistically significant, *P* value < 0.0042.

The median health system delay among TB patients was 34 days (IQR, 19–76 days). Patients seeking care at MMH at first consultation reported a significantly shorter health system delay as compared to the patients visiting other health care providers first (*P* = .001). A longer health system delay was found in patients with > 3 visits to health care providers compared to those with ≤ 3 visits (*P* < .001). The health system delay tended to be longer among respondents with higher educational level, TB lymphadenitis and who were HIV negative, as compared to those with lower educational level, other sites of TB infection and who were HIV positive, respectively, but without reaching statistical significance. Among patients first consulting other health facilities than MMH, the health system delay before referral to MMH was significantly longer than the delay at MMH (median, 22 vs. 11 days, *P* = .011).

The median total delay among TB patients was 62 days (IQR, 31–126 days), 15% had much longer total delay exceeding 6 months and 8% had total delay exceeding one year. Among the patients delaying > 6 months, 90% were diagnosed with TB lymphadenitis. The health system delay was significantly longer as compared to the patient delay *(P* < .001) and the greatest contributor to the total delay. In 80% of the patients the health system delay was longer than the patient delay. A longer total delay was noted for patients renting a house/living with relatives compared to patients owning their house, but the difference was not statistically significant. The only significant difference in total delay was found when comparing delay according to the site of TB disease, where a longer total delay was experienced by patients with TB lymphadenitis (P < .001), where 26% of the TB lymphadenitis patients faced a total delay exceeding 6 months.

### TB knowledge

Table 2 shows the level of TB knowledge among the TB and non-TB patients and S1 Table presents the distribution of answers to the various TB knowledge questions according to TB category, sex, HIV status and educational level. The knowledge about extrapulmonary site of TB was extremely poor. Fifty-five percent knew that the lungs could be affected by TB, as compared to only one patient who mentioned an extrapulmonary site. Most patients (93%) had heard of TB disease before being included in the study, and 73% answered respiratory and/or constitutional symptoms when asked about symptoms of TB disease. None of the respondents mentioned any other local symptoms than respiratory symptoms. Most of the respondents knew that TB is a curable disease (69%) and that TB can spread from person to person (79%). The median TB knowledge score was 5 (IQR, 3–6). The respondents with education above primary level had a higher TB knowledge score compared to patients with lower educational level (P = .007).

### Morbidity due to TB and improvement in self-rated health status after treatment

**Fig 2:**
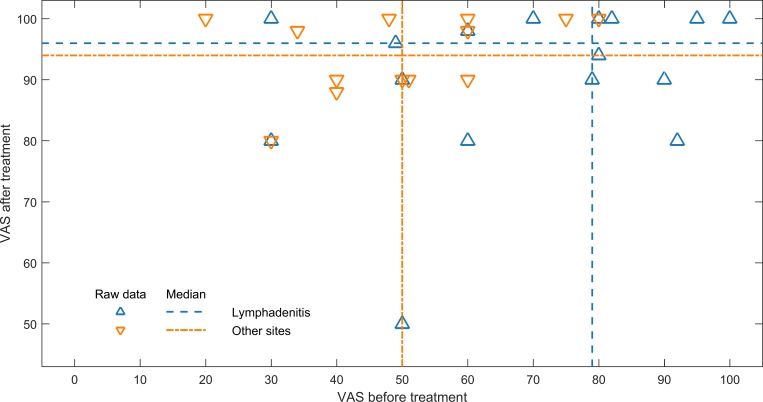
Scatterplot showing the EQ visual analogue scale scores before and after TB treatment. The scores are divided according to the site of TB infection, where the TB lymphadenitis patients are indicated with blue colour and other sites of TB infection with orange colour. The median scores in both groups before and after treatment are shown. Both groups reported significantly higher self-rated health after treatment.

**Fig 3:**
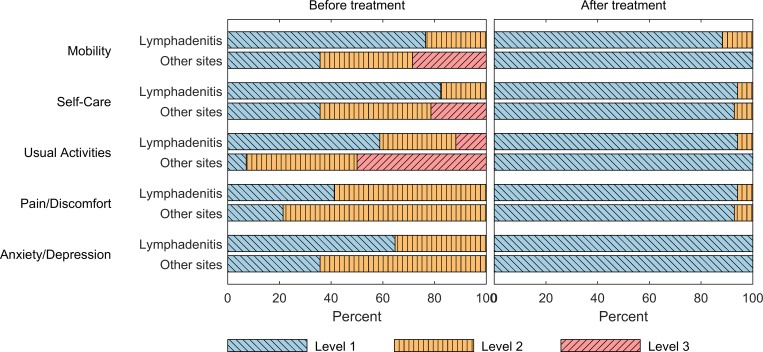
Stacked bar plot describing the patient statements in each of the 5 dimensions of the EQ-5D descriptive system before and after TB treatment. Level 1, no problems; level 2, some problems; level 3, extreme problems. Both groups reported lesser problems after treatment.

In the adult EPTB patients, 19/47 (40%) reported that they had completely stopped working and 20/47 (43%) reported reduced working capacity due to the current illness. Among respondents with TB lymphadenitis (n = 23), 70% reported reduced working capacity/stopped working, while this was noted in 96% of the respondents with other sites of TB infection (n = 24). The TB lymphadenitis patients reported a median of 90 days (IQR, 60–120 days) of reduced working capacity/stopped working, and patients with other sites of TB infection a median of 30 days (IQR, 14–60 days). Adult EPTB patients (n = 31) reported significantly higher self-rated health in the EQ VAS after compared to before ATT. The median EQ VAS score before and after ATT was 60% (IQR 48–80%) and 96% (IQR 90–100%) (*P* < .001), respectively. Respondents with TB lymphadenitis (n = 17) had a relatively better self-rated health before treatment, (EQ VAS; median, 79%, IQR, 50–91%) as compared to patients with other sites of TB infection (pleuritis, n = 10; peritonitis, n = 3; spondylitis, n = 1) (EQ VAS; median, 50%, IQR, 38.5–60%) (*P* = .015). Both groups reported significantly higher self-rated health after treatment as compared to before treatment (TB lymphadenitis, *P* = .002; other sites of TB infection, *P* = 0.001) (Fig 2). A similar pattern was observed when the health status was assessed with the EQ-5D descriptive system. Before treatment, patients with other sites of TB infection described more problems in all dimensions as compared to patients with TB lymphadenitis. Still, both groups reported lesser problems at the end of treatment than before treatment (Fig 3).

## Discussion

This study found that many EPTB patients, presenting to the main referral hospital in Zanzibar, experienced significant delay from the onset of symptoms until the start of TB treatment. Even though EPTB have been reported as a predictor of longer delays [14–16, 25–27], most earlier studies have been focusing on delay and associated factors in PTB, particularly smear-positive PTB. Cases of EPTB are seldom infectious and thus have less consequences for the spread of the TB disease, and probably therefore have not been studied specifically. However, for the patients, delay in the start of treatment may results in increased morbidity and mortality. A study from South-Africa [28], reported increased mortality when provider delay was ≥ 30 days. Further, TB poses an economic burden on affected patients and their household [29, 30]. In our study, 83% of the adult EPTB patients reported that they had completely stopped working or had reduced working capacity due to the current illness, a finding similar to a study from Kenya [30], where 85% of the respondents reported a decrease in the number of hours worked per week as a result of TB illness. Further, the adult EPTB patients in our study reported significantly higher self-rated health using the EQ VAS and described lesser problems in the EQ-5D descriptive system after TB treatment. Thus, this indicates that reducing the delay in the diagnosis and treatment of EPTB could decrease patient morbidity and have a positive impact on the economic situation for the patients and their families.

To the best of our knowledge there is no defined consensus on what constitutes acceptable and unacceptable delay in the diagnosis and initiation of treatment in EPTB patients. Some studies also including EPTB patients have used > 30 days and > 14 days as unacceptable patient and health system delay, respectively [17, 31]. Using these cut-offs, 76% of the EPTB patients in the present study reported to a health care provider within the acceptable patient delay, whereas only 17% had acceptable health system delay.

The total delay with a median of 62 days was found to be in agreement with previous reports including EPTB patients from resource-constrained settings [17, 25, 32], while other studies have found longer total delays [33]. Long total delays among EPTB patients have also been reported in industrialized countries [16, 27]. Previous studies have demonstrated that there is no consistent pattern of patient and health system delays between various settings. In the current study, health system delay was the major contributing factor to the total time to diagnosis and treatment, consistent with studies from settings as diverse as India [34], South Africa [28] and France [35]. Other studies from Nepal [32], Ethiopia [26] and Denmark [27] have reported patient delay as a major contributor towards total delay. These studies indicate that factors contributing to delay may vary depending on the setting and the study population.

The median patient delay of 14 days in our study was short compared to the patient delay reported in studies from Rwanda [25], Angola [36], Ethiopia [26] and mainland Tanzania [37], but longer than a study among children in Delhi, India [34]. These differences may be explained by the different distribution of urban and rural populations in the various studies, and in the availability of medical care. Unlike the findings reported from the United Kingdom [38] and Norway [14] we found no difference in patient delay according to age groups. In addition, no difference in patient delay was noted between men and women, a finding similar to Ethiopia [26], but different from South Africa [28], where a longer patient delay was reported among males. Self-treatment before consulting a health care provider was common in our study, but were not associated with longer patient delay, a finding different from a study from Ethiopia [15] reporting self-treatment as a predictor of patient delay. In our study few (10%) patients reported visiting a traditional healer, which may also be an explanation for the relative short patient delay, as consulting traditional healers is consistently reported to be associated with longer patient delay [12].

The median health system delay of 34 days was longer than acceptable and a significant contributor towards total delay in our study. This is comparable with delay reported in previous studies also including EPTB patients from Ethiopia [15], South Africa [28] and Rwanda [25], but longer than reported in another study from Ethiopia [26]. The shorter delay in the Ethiopian study could be explained by patients presenting with more advanced symptoms of disease since the median patient delay was 60 days in this study. Again, other studies from Norway [14] and United Kingdom [16] reported both longer patient and health system delay among EPTB patients than the present study, which could be because of a lower index of suspicion of TB in low-endemic countries. A long health system delay may partly be explained by the wide range of clinical presentation of EPTB disease, the low sensitivity of routine diagnostic methods such as acid-fast bacilli microscopy [39, 40] and mycobacterial culture [39, 41], lack of trained personnel and facilities for performing invasive procedures, thus increasing the difficulty in obtaining adequate biological samples for laboratory investigations, and the limited capacity for diagnostic imaging in low-resource settings. In addition, being dependent on patients returning for follow-up visits to receive results from various investigations or patients delaying consultation at a higher health care level even though a referral has been advised, could be contributing factors to the health system delay. A longer health system delay was found in patients with > 3 visits to a health care provider, which is in agreement with a study from India reporting that number of providers consulted until TB diagnosis was associated with health system delay [34]. As concluded in a review of delay in the diagnosis and treatment of tuberculosis, “The core problem in delay of diagnosis and treatment seemed to be a vicious cycle of repeated visits at the same healthcare level, resulting in nonspecific antibiotic treatment and failure to access specialized TB services” [11]. In our study, 48% of the TB patients reported > 3 visits and 38% ≥ 5 visits to a health care provider due to the current illness. Further, 81% reported repeated visits to MMH before diagnosis and initiation of treatment. In TB patients with initial visit to other health care providers than MMH, the health system delay prior to referral to MMH was longer as compared to the delay at MMH, and 47% reported repeated visits at the same level before referral to MMH. Advancing disease and the availability of diagnostic tools as imaging and laboratory investigations could have contributed to the shorter delay at MMH. At MMH, 37% of these patients were not started on ATT within 2 weeks of their first visit to the hospital. A high threshold for initiation of treatment, awaiting diagnostic proof, could also cause delay in the management of EPTB patient. On the other hand, starting treatment only based on clinical presumptive EPTB, leads to overtreatment. In our study, ATT was given to 19% of the non-TB cases, highlighting the importance of laboratory confirmation of EPTB and close follow-up of EPTB patients during treatment.

Patients with higher educational level tended to have longer health system delay, which is somewhat surprising. The explanation for this is uncertain, but there was a higher percentages of TB lymphadenitis cases among those with higher educational level compared to patients with lower educational level (56% vs 35%), which could explain the tendency for a longer health system delay in these patients.

In our study, patients with TB lymphadenitis experienced the longest median total delay of 99 days as compared to TB at other sites. A study from Tanzania also reported a long median diagnostic delay of 119 days (17 weeks) among TB lymphadenitis patients [36]. The indolent course of disease, lesser local symptoms, and fewer or no constitutional symptoms in lymphadenitis may be the reason for longer delay. Frequent involvement of cervical lymph nodes in different infections and lack of confirmatory TB tests may also have contributed towards the longer health system delay in lymphadenitis. Despite the indolent course, it is important to reduce the delay as these patients experienced significant improvement in self-rated health after treatment.

The level of TB knowledge in our study may be a reasonable indicator of the knowledge regarding TB in the general population in Zanzibar, since TB knowledge was assessed in both TB and non-TB patients and the TB patients were interviewed preferably before starting ATT, and by that they had not yet received the information following initiation of treatment. Our study indicates that while patients had relatively good knowledge of PTB, the knowledge regarding EPTB was very low, identifying a potential area of intervention.

Our study has some limitations. The samples size is small, limiting the possibility of performing further statistical analysis on factors associated with longer delays. The patients were enrolled only from the main referral hospital in Zanzibar, and patients initiated on treatment at the primary or secondary care level are not represented in the study. Thus, the results may not necessarily be generalisable to all patients presenting with presumptive EPTB to the health care system in Zanzibar. The information regarding symptoms, onset of symptoms and health care seeking behaviour are mainly self-reported and implies the possibility of recall bias. We did not record the patients visit to over-the-counter drug shops, and there is a possibility that some of the patient first sought care at these sites, further reducing the actual patient delay. Further, the study participants were part of a study implementing and validating a new diagnostic test for EPTB at MMH, and being part of this study may potentially have influenced health system delay in either direction. Finally, use of a composite reference standard for categorization of TB patients is not perfect and some patients with other chronic bacterial infections could have responded to the ATT and wrongly categorized as TB patients. Similarly, inadequate response due to poor compliance or drug resistance could have led to wrong categorization of patients as non-TB cases. However, subanalysis of the bacteriologically or clinically confirmed TB groups did not show difference in various outcomes (data not presented due to small sample size), and unlikely to have an impact on the conclusions drawn in this study.

## Conclusion

Many EPTB patients presenting to the main referral hospital in Zanzibar had delay in the diagnosis and treatment exceeding two months, and the greatest proportion of this delay occurred due to the health system delay. The self-rated health among adult EPTB patients was significantly higher after treatment, implying that appropriate and timely treatment of EPTB disease have the potential to reduce morbidity and the economic loss for the patient and their families. A reduction of diagnostic delay could be achieved through strengthening the knowledge and awareness of EPTB in the general population by incorporating information on EPTB in public health educational campaigns, continuous training of all health care providers, at all levels, in early recognition of symptoms suggestive of EPTB and the diagnostic possibilities, proper information and a scheduled follow-up of patients receiving a trial of antibiotics and strengthening the collaboration between the national TB programme and private and public health facilities such as outpatient clinics and hospital wards performing invasive diagnostic procedures. Further, since most peripheral health units do not have diagnostic facilities for diagnosing EPTB it could be feasible to establish algorithms of timely referral of presumptive EPTB patients to health facilities with diagnostic capacity and higher medical expertise to shorten the care-seeking pathway. Finally, support and strengthening of the laboratories performing TB diagnostics services is of utmost importance.

## Supporting information

S1 Table Distribution of answers to TB knowledge questions according to TB category, sex, HIV status and educational level. (DOCX)

S1 File Data set. (SAV)
