# Supplementary material for: Diagnostic delay in extrapulmonary tuberculosis and impact on patient morbidity: A study from Zanzibar
Source: PLoS One. 2018 Sep 6;13(9):e0203593. doi: 10.1371/journal.pone.0203593 (PMC6126857; doi:10.1371/journal.pone.0203593)
Supplement: S1 Table — (DOCX) [file pone.0203593.s001.docx]

| S1 Table Distribution of answers to TB knowledge questions according to TB category, sex, HIV status and educational level | | | | | | | | | |
| --- | --- | --- | --- | --- | --- | --- | --- | --- | --- |
|  |  | **TB category^a^** | | **Sex^b^** | | **HIV status^c^** | | **Educational level^d^** | |
|  |  | **TB patient**  *n* = 68 | **Non-TB patient**  *n* = 63 | **Male**  *n* = 45 | **Female**  *n* = 43 | **HIV negative**  *n* = 77 | **HIV positive**  *n* = 21 | **≤ primary school**  *n* = 46 | **˃ primary school**  *n* = 41 |
| Question |  | *n* (%) | *n* (%) | *n* (%) | *n* (%) | *n* (%) | *n* (%) | *n* (%) | *n* (%) |
| 1. Ever heard of TB? | Yes | 63 (93%) | 59 (94%) | 42 (93%) | 40 (93%) | 72 (94%) | 19 (90%) | 42 (91%) | 39 (95%) |
|  | No | 5 (7%) | 4 (6%) | 3 (7%) | 3 (7%) | 5 (6%) | 2 (10%) | 4 (9%) | 2 (5%) |
| 2. Do you know any TB symptoms? | Only RS | 27 (40%) | 35 (56%) | 18 (40%) | 20 (47%) | 34 (44%) | 6 (29%) | 19 (41%) | 19 (46%) |
|  | Only CS | 1 (1%) | 2 (3%) | 3 (7%) | 0 | 0 | 3 (14%) | 0 | 3 (7%) |
|  | RS and CS | 18 (26%) | 12 (19%) | 7 (16%) | 14 (33%) | 18 (23%) | 7 (33%) | 9 (20%) | 12 (29%) |
|  | No | 22 (32%) | 14 (22%) | 17 (38%) | 9 (21%) | 25 (32%) | 5 (24%) | 18 (39%) | 7 (17%) |
| 3. Knowledge about EPTB: which parts of the body could be affected by TB? | Lungs | 37 (54%) | 35 (56%) | 24 (53%) | 24 (56%) | 40 (52%) | 15 (71%) | 22 (48%) | 26 (63%) |
|  | Lungs and another site | 1 (1%) | 0 | 0 | 1 (2%) | 1 (1%) | 0 | 0 | 1 (2) |
|  | Don`t know | 30 (44%) | 28 (44%) | 21 (47%) | 18 (42%) | 36 (47%) | 6 (29%) | 24 (52%) | 14 (34%) |
| 4. TB spread: person to person? | Yes | 54 (79%) | 50 (79%) | 37 (82%) | 35 (81%) | 60 (78%) | 19 (90%) | 34 (74%) | 38 (93%) |
|  | No/uncertain | 14 (21%) | 13 (21%) | 8 (18%) | 8 (19%) | 17 (22%) | 2 (10%) | 12 (26%) | 3 (7%) |
| 5. Can TB spread from animals to humans? | Yes | 8 (12%) | 10 (16%) | 9 (20%) | 6 (14%) | 9 (12%) | 5 (24%) | 7 (15%) | 8 (20%) |
|  | No | 60 (88%) | 53 (84%) | 36 (80%) | 37 (86%) | 68 (88%) | 16 (76%) | 39 (85%) | 33 (80%) |
| 6. Curable with medicine? | Yes | 47 (69%) | 43 (68%) | 32 (71%) | 32 (74%) | 53 (69%) | 15 (71%) | 32 (70%) | 31 (76%) |
|  | No/uncertain | 21 (31%) | 20 (32%) | 13 (29%) | 11 (26%) | 24 (31%) | 6 (29%) | 14 (30%) | 10 (24%) |
| 7. Duration of ATT | Yes | 27 (40%) | 18 (29%) | 14 (31%) | 18 (42%) | 23 (30%) | 13 (62%) | 15 (33%) | 17 (41%) |
|  | No | 41 (60%) | 45 (71%) | 31 (69%) | 25 (58%) | 54 (70%) | 8 (38%) | 31 (67%) | 24 (59%) |

**Abbreviations:** TB, tuberculosis; HIV, human immunodeficiency virus; RS, respiratory symptoms (coughing, productive cough, haemoptysis, shortness of breath, chest pain); CS, constitutional symptoms (fever, weight loss, night sweat, tiredness/fatigue, reduced appetite); EPTB, extrapulmonary tuberculosis; ATT, antituberculosis treatment

^a^ Patients with missing values excluded (n=1).

^b^ Only patients ≥ 15 years (n=89). Patients with missing values excluded (n=1).

^c^ Only patients with known HIV status (n=99). Patients with missing values excluded (n=1).

^d^ Only patients ≥ 15 years (n=89). Patients with missing values excluded (n=2).

**Note: Scoring system for calculating the TB knowledge score:** Question 1, 4, 5, 6, 7: 1 point for each “correct” answer; Question 2: 1 point if 1 of the following and 2 points if ≥2 of the following - respiratory symptom(s), constitutional symptom(s), other local symptom(s); Question 3: 1 point if answering lungs, 2 points if answering lungs and another site. Maximum score was 9.
